# Mammalian Mon2/Ysl2 regulates endosome-to-Golgi trafficking but possesses no guanine nucleotide exchange activity toward Arl1 GTPase

**DOI:** 10.1038/srep03362

**Published:** 2013-11-28

**Authors:** Divyanshu Mahajan, Boon Kim Boh, Yan Zhou, Li Chen, Tobias Carl Cornvik, Wanjin Hong, Lei Lu

**Affiliations:** 1 School of Biological Sciences, Nanyang Technological University, 60 Nanyang Drive, Singapore 637551; 2 Institute of Molecular and Cell Biology, 61 Biopolis Drive, Proteos, Singapore 138673

## Abstract

Arl1 is a member of Arf family small GTPases that is essential for the organization and function of Golgi complex. Mon2/Ysl2, which shares significant homology with Sec7 family Arf guanine nucleotide exchange factors, was poorly characterized in mammalian cells. Here, we report the first in depth characterization of mammalian Mon2. We found that Mon2 localized to *trans*-Golgi network which was dependent on both its N and C termini. The depletion of Mon2 did not affect the Golgi localized or cellular active form of Arl1. Furthermore, our in vitro assay demonstrated that recombinant Mon2 did not promote guanine nucleotide exchange of Arl1. Therefore, our results suggest that Mon2 could be neither necessary nor sufficient for the guanine nucleotide exchange of Arl1. We demonstrated that Mon2 was involved in endosome-to-Golgi trafficking as its depletion accelerated the delivery of furin and CI-M6PR to Golgi after endocytosis.

Arf family small GTPases are known to play essential roles in membrane trafficking^1^,^2^. Like other small GTPases, Arfs cycle between inactive GDP and active GTP bound form. The cycling is catalyzed by guanine nucleotide exchange factors (GEFs) and GTPases activating proteins (GAPs). For example, GDP bound Arf1 (Arf1-GDP) is cytosolic but once the bound GDP is exchanged to GTP by Arf GEFs, it associates with the Golgi membrane and recruits effectors to modify membrane lipids and control vesicular coat recruitment^2^. Arf GEFs consist of diverse members with molecular weights ranging from 40 to 200 kd. All Arf GEFs share a ~200 amino acids Sec7 domain, which is necessary and sufficient for the guanine nucleotide exchange activities of Arf GEFs in vitro^3^. The GBF/BIG family Arf GEFs are Golgi localized large molecular weight proteins (170–200 KDa), which are conserved from yeast to mammal. Members of this family are GBF1, BIG1/2 in mammal and Gea1/2p and Sec7p in yeast. Outside the Sec7 domain, additional five domains are shared among members, including DCB (dimerization and cyclophilin binding), HUS (homology upstream of Sec7) and HDS1-3 (homology downstream of Sec7) domains^4^.

Recently, the Arf family expanded greatly by the inclusion of many Arf-like proteins (Arls)^5^. Arl1 is the best studied among Arls and it is known to play essential roles in the organization of Golgi and membrane trafficking between endosome and *trans*-Golgi network (TGN)^6^,^7^. Arl1-GTP recruits Golgins, such as Golgin97 and Golgin245 by specifically interacting with the GRIP (Golgin-97, RanBP2α, Imh1p and p230/Golgin-245) domain^8^,^9^. We know little about the regulation of Arl1 compared to Arfs. In yeast, the Arf GAP Gcs1p could terminate the active Arl1p and return it to inactive form^10^. Imaging data demonstrated that the Golgi association of Arl1p, which indicates the activation of Arl1p, was regulated by Sys1p and Arl3p/ARFRP1 in yeast and mammalian cells^11^,^12^,^13^. Two GEFs for Arl1 have been reported so far – Syt1p and Mon2p. Syt1p is a Sec7 domain containing protein that has GEF activity for Arf1/2p and Arl1p^14^,^15^. In yeast, in vivo and in vitro data showed that the activation of Arl1p requires Syt1p^15^. However, Syt1 is unique to fungi and, therefore Mon2, which is conserved in eukaryotes, remains the only known candidate GEF for Arl1 in metazoan.

Mon2 was independently identified in yeast through genetic screenings for monensin and Brefeldin A (BFA) resistance, endocytosis and vacuole defects, and synthetic lethality with *Δypt51*^16^,^17^,^18^,^19^,^20^. Yeast Mon2p was reported to regulate endosome-to-late Golgi trafficking, endocytosis, vacuole biogenesis and cytosol to vacuole (cvt) pathway^21^,^22^,^23^. Mon2p was found to interact with Dop1p^22^,^23^,^24^, Neo1p^25^ and GGA1^26^. Evidence supporting its role as Arl1 GEF was mainly based on three findings. First, yeast and human Mon2 sequences are related to GBF/BIG family Arf GEFs and they share DCB, HUS and HDS1-3 domains^21^,^22^,^23^. Second, Mon2p was reported to have a Sec7 homologous region, where Arl1p-GDP preferentially interacted in vitro^21^. Third, Arl1p and Mon2p had genetic interactions in yeast^21^. However, later studies pointed out that Mon2 does not have a significant Sec7 domain^22^,^23^ and the activation of Arl1 seemed to be normal in *ΔMon2* yeast^11^,^13^,^22^. Furthermore, a recent study implied that Mon2p could be a negative regulator of Arl1^27^. Together, these studies suggested Mon2 could not be the GEF for Arl1. Here, we attempted to initially characterize the mammalian Mon2 and further test if Mon2 could be a GEF for Arl1. We found that Mon2 localized to the TGN and it was involved in endosome-to-Golgi trafficking. Our in vivo and in vitro data argue that Mon2 could not be a GEF for Arl1.

## Results

### Mammalian Mon2 is localized to the *trans*-Golgi network

**Figure 1:**
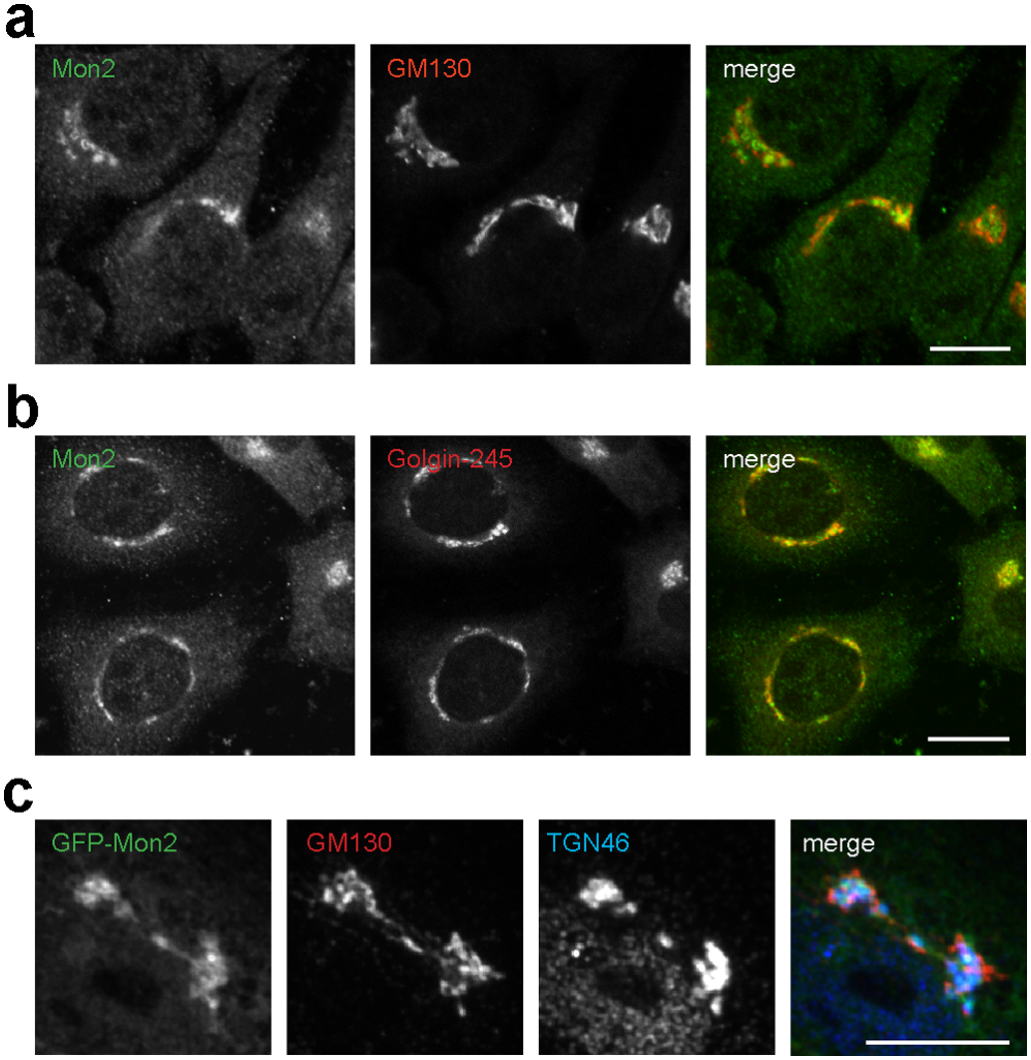
Mammalian Mon2 is localized to the *trans*-Golgi network (TGN). (a) HeLa cells were labeled by antibodies against Mon2 and GM130. (b) HeLa cells were labeled by antibodies against Mon2 and Golgin245. (c) BSC1 cells were transfected by EGFP-Mon2 and labeled by antibodies against GM130 and TNG46. Mon2 colocalized with TGN46, a TGN marker, more than GM130, a *cis*-Golgi marker. Bars, 10 μm.

In *Saccharomyces cerevisiae*, tagged Mon2p localizes at late Golgi compartment^22^,^23^. However, the sub-cellular localization of mammalian Mon2 is still unclear. To that end, we raised a rabbit polyclonal antibody against human Mon2. The purified antibody is able to recognize and immunoprecipitate endogenous and exogenously expressed Mon2 in Western blot (Sup. Fig. 1 a and b). In indirect immunofluorescence, our Mon2 polyclonal antibody showed a perinuclear staining pattern, colocalizing with Golgi markers such as GM130 and Golgin245 (Fig. 1 a and b) in HeLa cells. This perinulcear staining pattern disappeared when the antibody was neutralized by its antigen or Mon2 was knocked-down by siRNA (Sup. Fig. 1 c and d), indicating our antibody specifically labels endogenous Mon2. We cloned the EGFP and Myc-tagged full length of human Mon2 and found that both colocalized with Golgi markers, such as Arl1 (Sup. Fig. 1e), GM130 and TGN46 (Fig. 1c), indicating that exogenous expressed Mon2 is also correctly targeted to the Golgi. Close inspection of the Golgi distribution patterns revealed that Mon2 colocalized more with TGN markers, such as Golgin-245 and TGN46, than *cis*-Golgi ones, such as GM130 (Fig. 1c), therefore indicating Mon2 preferentially associates to the TGN in mammalian cells. Similar localization results were also observed in other cultured cell lines, such as BSC1, RPE1, NRK and C2C12 cells via EGFP-tagged or endogenous Mon2 (data not shown).

**Figure 2:**
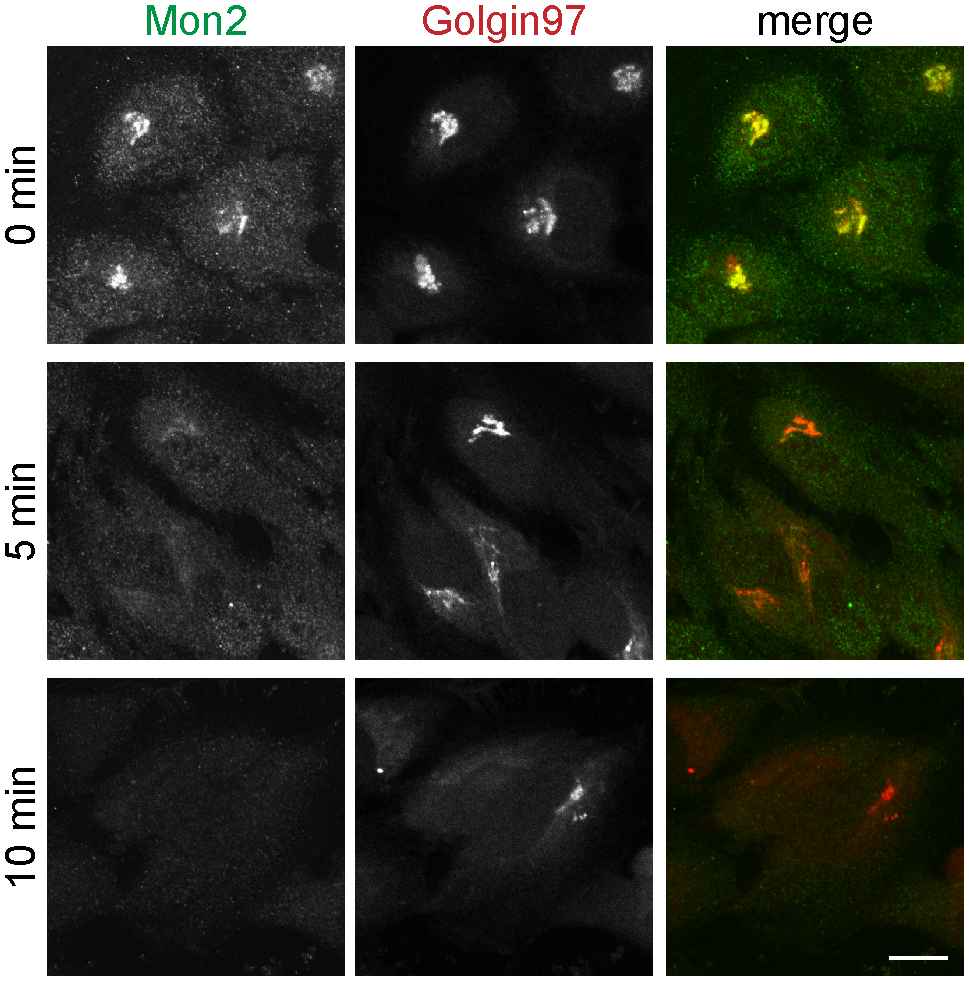
Mon2 dissociates from Golgi faster than Golgin97 under BFA treatment. HeLa cells were treated with 10 μg/ml BFA for 0 min (upper panel), 5 min (middle panel) and 10 min (lower panel). Cells were labeled by antibodies against Mon2 and Golgin97. Bar, 10 μm.

Brefeldin A (BFA) is a fungal metabolite that is capable of rapidly inactivating and dissociating Arf1 and subsequently causing the disassembly of the Golgi complex^28^. Many Golgi localized peripheral membrane proteins lose their Golgi associations at different kinetics. While ARF1 and its effectors, such as COPI, AP1 and GGAs, dissociate from Golgi within 5 min of BFA treatment^29^, we previously showed that Arl1 and its effectors, such as Golgin97 and Golgin245, do so after 10 min of treatment^6^,^9^. When HeLa cells were treated by BFA for 5 min, both endogenous Mon2 and GGA2 lost their perinuclear staining (Sup. Fig. 2) although a significant amount of Golgin97 was still observed at the perinuclear region (Fig. 2). Therefore, similar to Arf1 and its effectors, the Golgi localization of Mon2 is more sensitive to BFA than Arl1 and its effectors. This result indicates that Golgi localization of Arl1 and its effectors is independent of Mon2. Collectively, our data demonstrate that mammalian Mon2 resides at the TGN, in agreement with the late Golgi localization of its yeast ortholog, but its dissociation from the Golgi by short BFA treatment has no significant impact on the Golgi localization of Arl1.

### Golgi localization of Mon2 requires both N and C - termini

**Figure 3:**
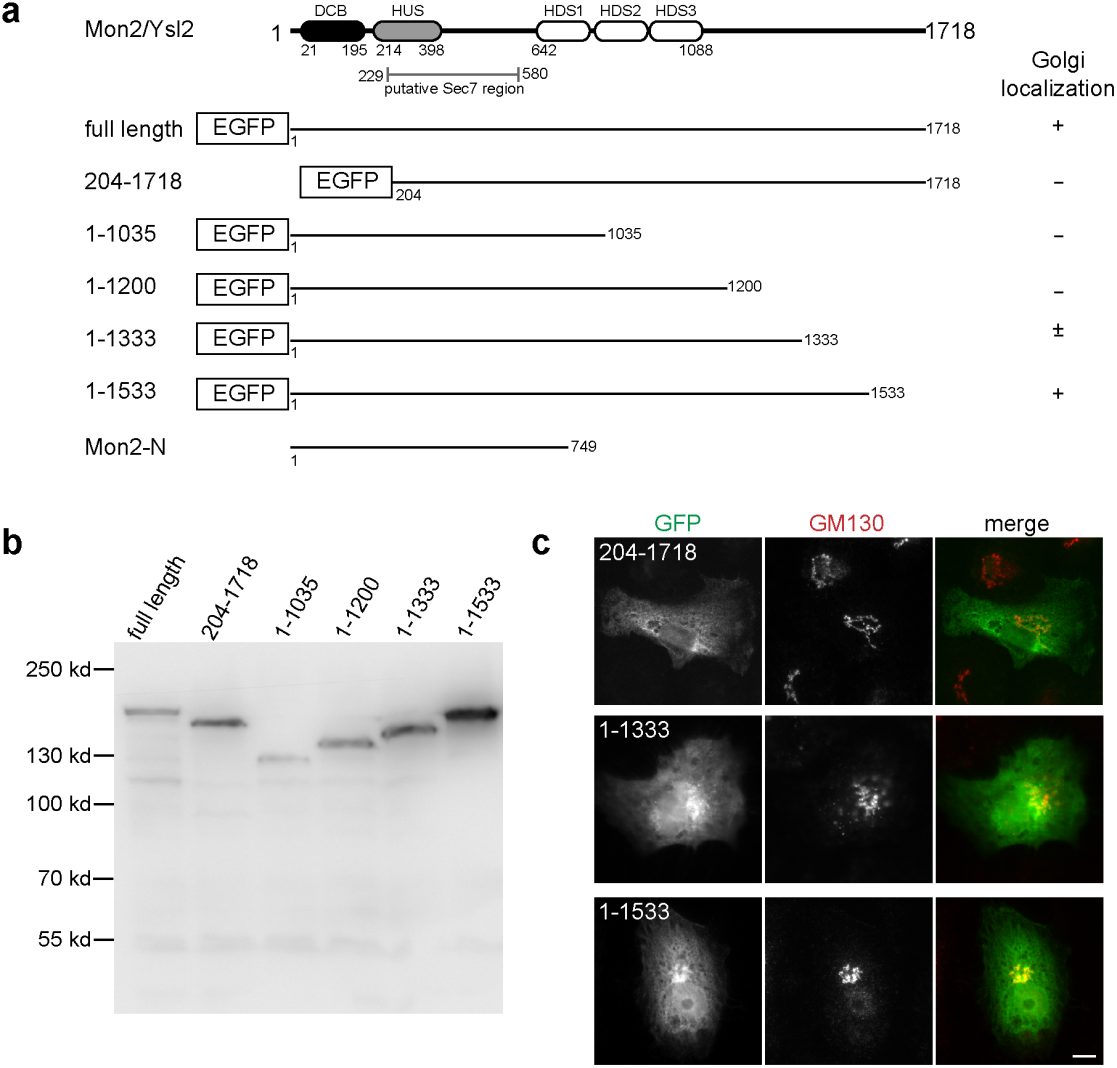
The Golgi association of Mon2 requires both N and C-termini. (a) Schematic diagram showing the domain organization of human Mon2 and the alignment of various Mon2 truncation clones. The DCB, HUS and HDS1-3 domains were identified through PSI-BLAST. The putative Sec7 region, which was previously reported in yeast, was identified by subjecting human and yeast Mon2 sequences to BLAST. The result of Golgi localization is summarized at right. +, Golgi localization positive; −, Golgi localization negative; ±, weak Golgi localization. (b) Mon2 truncation clones expressed truncated proteins at expected sizes. HeLa cells were transfected with respective EGFP-tagged Mon2 truncation clones. The resulting cell lysates were separated in SDS-PAGE and blotted by GFP antibody. (c) Selected images of EGFP-Mon2 truncation clones. BSC1 cells transfected with EGFP-Mon2 truncation clones were labeled by anti-GM130 antibody. Bar, 10 μm.

Yeast and human Mon2 sequences are distantly related to Arf GEFs^21^,^22^,^23^ and the five conserved domains among Arf GEFs (Fig. 3a), including DCB, HUS and HDS1-3^4^, could also be identified in yeast and human Mon2 as noted previously^22^,^23^. To determine which domains are essential for the Golgi targeting of Mon2, EGFP fused N and C - terminal serial truncations were made (Fig. 3a). All truncation clones expressed EGFP fusion proteins at expected molecular weights (Fig. 3b). When expressed in BSC1 cells (Fig. 3c), the DCB domain truncated Mon2 (clone 204–1718) lost its Golgi localization. However, the Golgi localization of Mon2 could tolerate the truncation of its C-terminus up to amino acid 1533 (clone 1–1533). Further deletion at C-terminus greatly weakened (clone 1–1333) (Fig. 3c) or abolished (clone 1–1200 and 1–1035) (Sup. Fig. 3) the Golgi localization. Therefore our data indicate that both N and C-termini of Mon2 are essential for its Golgi localization. In contrast, the N-terminal region of yeast Mon2p, including the DCB and HUS domain, was reported to be necessary and sufficient for Golgi targeting^23^.

### Depletion of Mon2 does not affect the localization of Arl1

**Figure 4:**
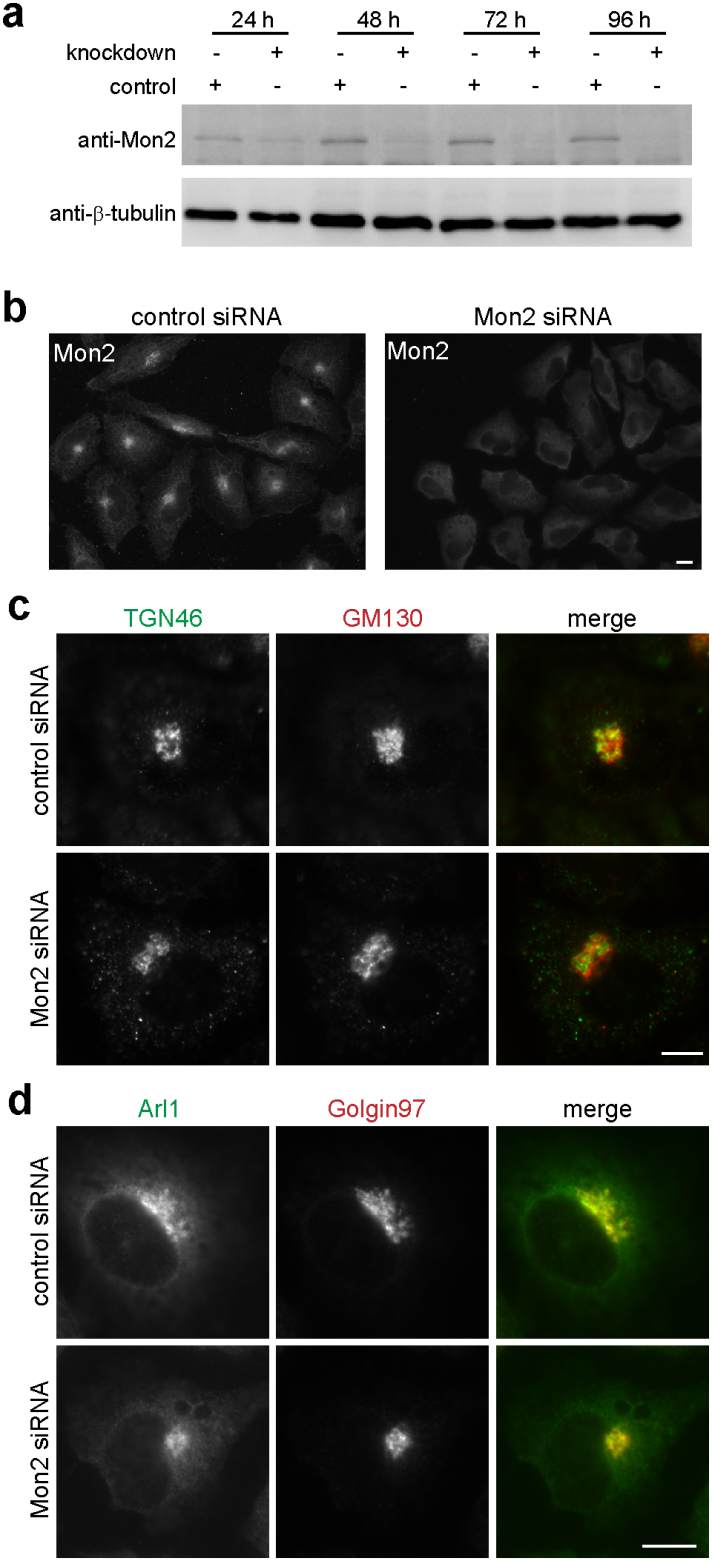
Depletion of Mon2 does not significantly affect the morphology of Golgi complex and the localization of Arl1. (a) Western blot showed that endogenous Mon2 level is significantly reduced under Mon2 siRNA treatment. HeLa cells were transfected by control or Mon2 specific siRNA for 24, 48, 72 and 96 hours. The cell lysate was separated in SDS-PAGE and blotted by anti-Mon2 and β-tubulin antibodies. β-tubulin served as a loading control. The images were cropped to highlight the regions of interest and the original gel blots are presented in Sup. Fig. 6a. (b) Immunofluorescence showed that endogenous Mon2 level is significantly reduced under Mon2 siRNA treatment. HeLa cells were treated by control or Mon2 specific siRNA for 96 hours and subsequently labeled by anti-Mon2 antibody. (c) Depletion of Mon2 did not significantly affect the morphology of Golgi apparatus. Mon2 depleted HeLa cells were labeled by antibodies against GM130 and TGN46. (d) Depletion of Mon2 did not affect the Golgi association of Arl1 and Golgin97. Mon2 depleted HeLa cells were labeled by antibodies against Arl1 and Golgin97. Bars, 10 μm.

Mon2 has been proposed to be a GEF for Arl1^21^. However, the hypothesis has not been systematically tested. If Mon2 plays a dominant role for the guanine nucleotide exchange of Arl1, Arl1-GTP would be greatly reduced upon depletion of Mon2 by siRNA mediated knockdown. In HeLa cells, we found that the endogenous Mon2 protein could be knocked-down with the incubation of its siRNA (Fig. 4a). After 96-hour treatment of siRNA, endogenous Mon2 protein could not be detected under Western blot and immunofluorescence (Fig. 4 a and b). Therefore, 96-hour (4-day) knockdown protocol was adopted for subsequent experiments.

When endogenous Mon2 was efficiently depleted, we found that the morphology of Golgi was largely unaffected except that it appeared more compact (Fig. 4c). *cis*-Golgi (GM130) and TGN markers (TGN46) were localized to tightly adjacent but non-overlapping structures in both knockdown and control cells, demonstrating that the *cis-trans* polarity of Golgi stack was not abolished in the absence of Mon2. Most importantly, Arl1 and its effector–Golgin97–were still observed in the Golgi similar to control cells (Fig. 4d). Our observation showed that Arl1 was activated normally in the absence of Mon2, therefore suggesting that Mon2 could not be essential for the guanine nucleotide exchange of Arl1. The finding is in agreement with our observation made during BFA treatment (see above).

### Depletion of Mon2 does not significantly change the quantity of active Arl1

**Figure 5:**
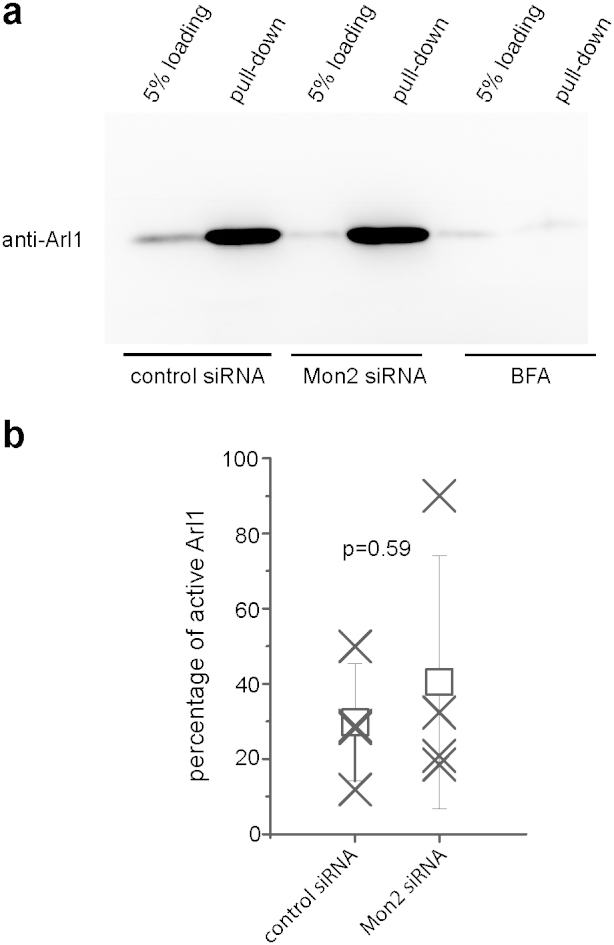
Depletion of Mon2 does not change the amount of active Arl1. (a) HeLa cells were subjected to control or Mon2 siRNA treatment for 96 hours. Cells were subsequently lysed and incubated with GST-GRIP protein immobilized on beads. The active Arl1 pulled-down was detected by Western blot and quantified. BFA is known to inactivate Arl1 after 30 min treatment and served as a negative control. The image was cropped to highlight the region of interest and the original gel blot is presented in Sup. Fig. 6b. (b) The percentage of active Arl1 in control and Mon2 siRNA treated HeLa cells showed no significant difference (p = 0.59) from 4 independent experiments (n = 4).

To monitor the amount of active Arl1 quantitatively under the depletion of Mon2, we adopted our previously developed assay to quantify the endogenous Arl1-GTP^30^. The assay is based on the highly selective and quantitative binding between GRIP domain and Arl1-GTP. Briefly, glutathione S-transferase (GST) fused GRIP domain of Golgin-245 was immobilized onto glutathione agarose beads. When the beads were incubated with the cell lysate from control or Mon2 knockdown cells, Arl1-GTP, instead of Arl1-GDP, was selectively pulled-down and quantified in Western blot using Arl1 antibody. A typical result is shown in Fig. 5a. As we previously demonstrated that prolonged treatment (30 min) of BFA inactivates endogenous Arl1^6^, BFA treated cells served as a negative control here and, as expected, less than 5% Arl1 was pulled-down (Fig. 5a). From four independent assays, the percentage of active Arl1 in control and Mon2 depleted cells was found to be 30 ± 16% and 40 ± 34% (mean ± SD; n = 4), respectively, which is not statistically significant by t-test (p = 0.59) (Fig. 5b). Our data indicate that depletion of Mon2 does not significantly change the amount of active Arl1 and therefore suggest that Mon2 is not essential for the activation of Arl1.

### The N-terminal region of Mon2 containing the putative Sec7 domain does not promote guanine nucleotide exchange of Arl1 in vitro

**Figure 6:**
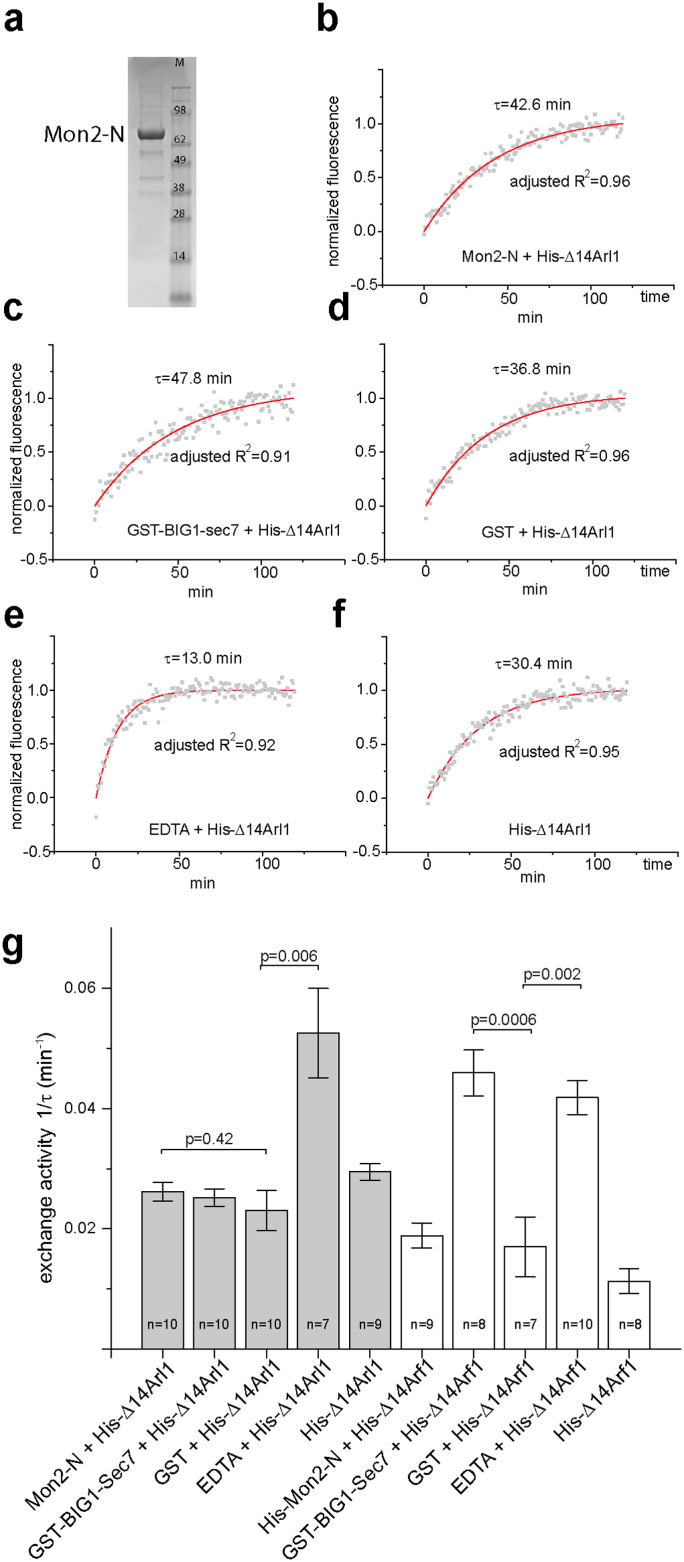
The putative Sec7 region of Mon2 does not promote guanine nucleotide exchange of Arl1 in vitro. (a) Coomassie stained gel showing the Mon2-N protein purified from insect cells. M, molecular weight marker (kd). (b–f) Typical exchange kinetic traces during in vitro guanine nucleotide exchange of Arl1. His-Δ14Arl1 (1.0 μM) was incubated with Mon2-N (0.7 μM) (b), GST-Big1-Sec7 (0.7 μM) (c), GST (0.7 μM) (d), EDTA (0.7 μM) (e) or buffer (f), respectively, in the presence of Mant-GMPPNP. The experimental data (gray squares) were fitted to a single exponential decay function y = y_0_ + A*exp[−(x − x_0_)/τ] (red curve) and normalized. The time constant τ and adjusted R^2^ are labeled in each plot. (g) The column graph showing the guanine nucleotide exchange activities (1/τ) of various factors on Arl1 or Arf1. n denotes the number of independent experiments. Error bar indicates standard error of mean. The p values (by t-test) of selected pair of data are indicated.

The N-terminus of yeast Mon2p, including HUS domain and a downstream region, was reported to be homologous to Sec7 domain and proposed to catalyze the guanine nucleotide exchange of Arl1p^21^. To explore if human Mon2 possesses that exchange activity, we identified the corresponding putative Sec7 region by aligning human and yeast Mon2 through two-sequence BLAST (Fig. 3a). We directly tested if the N-terminal region of Mon2, including its putative Sec7 domain, has in vitro guanine nucleotide exchange activity toward Arl1. In this in vitro assay, the guanine nucleotide exchange of small GTPase was monitored by the use of Mant-GMPPNP (guanosine 5′-[β,γ-imido]triphosphate), a non-hydrolyzable and fluorescent analog of GTP, whose quantum yield increases significantly upon binding to GTPase. Two reactions were introduced here as positive controls. The first one was the exchange of Arf1 catalyzed by the Sec7 domain of BIG1 (189 aa), which was reported to possess a low but significant GEF activity toward Arf1^31^. The second one was the exchange of Arf1 and Arl1 by EDTA, which catalyzes the exchange on most small GTPases by chelating their bound Mg^2+^. GST protein was used as a negative control. To facilitate in vitro exchange reaction, the N-terminal amphipathic α-helix of Arl1 or Arf1 was truncated^32^,^33^. The resulting His-Δ14Arl1 and His-Δ14Arf1 proteins, together with GST and GST-BIG1-Sec7, were purified from bacteria (Sup. Fig. 4a). The N-terminal region of Mon2 from amino acid 1 to 749 (Mon2-N) (schematically illustrated in Fig. 3a), comprising the putative Sec7 domain, was purified from insect cell (Fig. 6a).

In our in vitro assays, His-Δ14Arl1 or His-Δ14Arf1 was incubated with different testing factors – Mon2-N, GST-BIG1-Sec7, GST, EDTA or buffer – in the presence of Mant-GMPPNP at 25 °C. The guanine nucleotide exchange of Arl1 or Arf1 was registered as the increase of fluorescence intensity. As shown in typical exchange traces (Fig. 6b–f and Sup. Fig. 4b), different factors resulted in different kinetics of fluorescence increases. We found that the fluorescence intensity vs time traces could be nicely fitted by a single exponential function y = y_0_ + A*exp[−(x − x_0_)/τ] with adjusted R^2^ ≥ 0.90. The advantage of such curve fitting is that the exchange kinetics could be mathematically described by the inverse of time constant − τ. Since the final concentrations of testing factors and small GTPases remained constant in our assays, the 1/τ value, which is referred to as exchange activity, reflected the intrinsic exchange activity of a putative exchange factor. Similar data analysis approach was also used in a recent report^34^. The exchange activities of various factors from more than seven independent assays are shown in Fig. 6g. As expected, the exchange activity of EDTA on Arf1 and Arl1 was significantly higher than GST (p = 0.006 and 0.002, respectively, by t-test). In agreement with previous report on the exchange activity of Sec7 domain toward Arf1^31^, the exchange activity of GST-BIG1-Sec7 on Arf1 is significantly higher than that of GST (p = 0.0006). However, Mon2-N did not exhibit significantly more exchange activity toward Arl1 than GST (p = 0.42). Our data also showed that GST-BIG1-Sec7 had no exchange activity toward Arl1, suggesting a significant difference between Arf1 and Arl1 in term of guanine nucleotide exchange. In summary, our in vitro data indicate that Mon2 does not possess guanine nucleotide exchange activity toward Arl1, therefore arguing against the hypothesis that Mon2 is a GEF for Arl1. This conclusion is consistent with our BFA treatment and knockdown experiments (see above).

### Depletion of Mon2 accelerates the endosome-to-TGN trafficking of CD8A fused furin and CI-M6PR

In yeast, the deletion of Mon2 affects the appearance of endosome and disrupts the localization patterns of Golgi SNAREs such as Tlg2p and Snc1p^22^,^23^. Genetic data from worm implicated Mon2 in the retrograde trafficking from endosome to Golgi to maintain the cortical localization of β-catenin^35^. In mammalian cells, the knockdown of Mon2 was found to alter the distribution of early and recycling endosome marker – transferrin receptor^26^. However, it is unknown if mammalian Mon2 is also involved in endocytic trafficking similar to its yeast and worm ortholog. To that end, we created two reporter proteins which consist of luminal and transmembrane domain of CD8A and the C-terminal cytosolic domain of furin or cation-independent mannose 6-phosphate receptor (CI-M6PR). CD8A, furin and CI-M6PR are type I transmembrane proteins. CD8A, which is not present in commonly used cell lines, has been commonly utilized for studying the intracellular trafficking of type I transmembrane proteins. These CD8A chimeras would have trafficking itineraries and kinetics primarily determined by the sorting signals within the cytosolic domains of furin and CI-M6PR^36^. Following endocytosis from plasma membrane, CD8A fused CI-M6PR and furin are expected to be transported to TGN via early/recycling and late endosome, respectively, before reaching steady state in which both are primarily localized to the TGN and late endosomes^37^,^38^.

**Figure 7:**
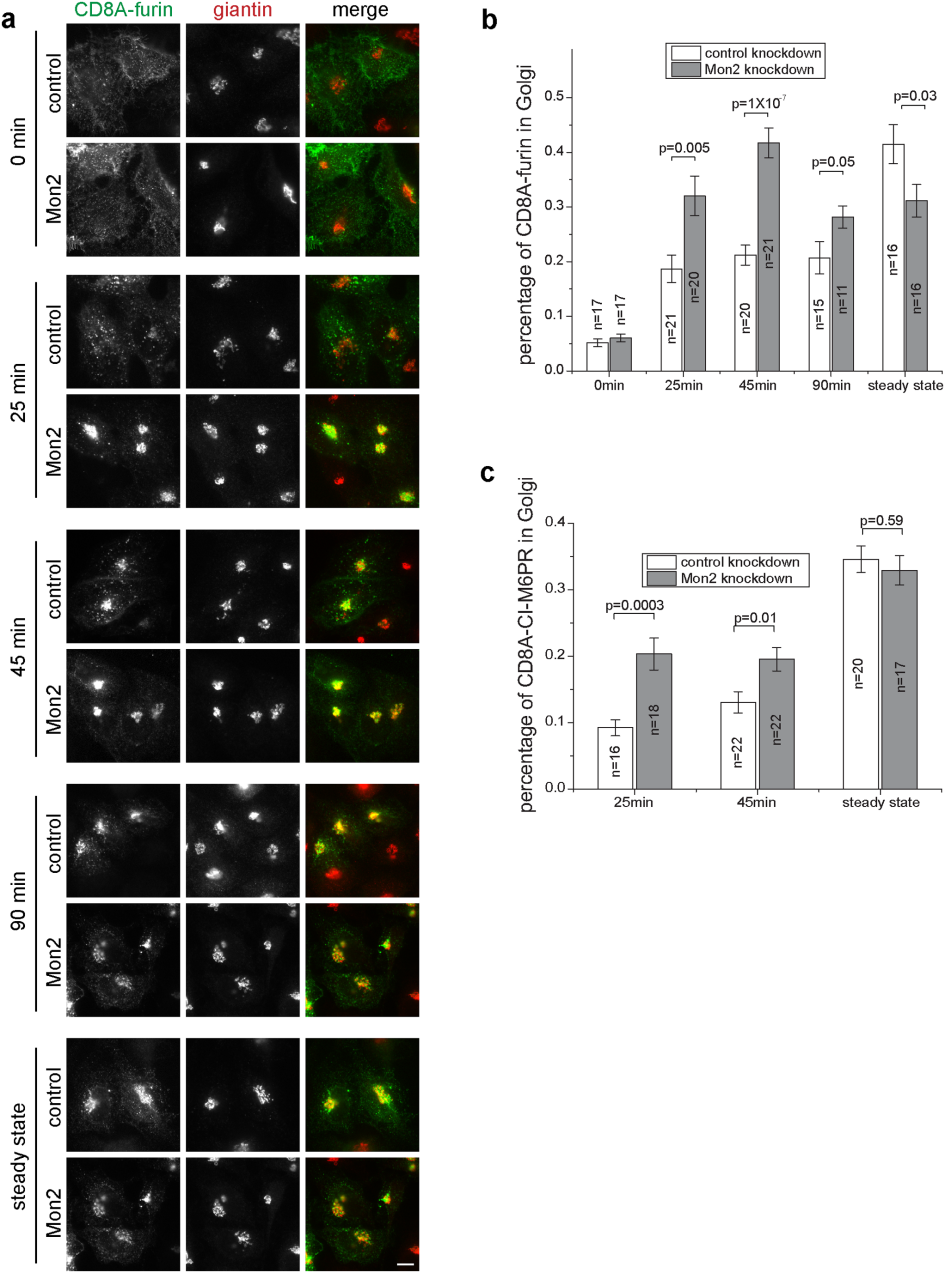
Depletion of Mon2 accelerates the endosome-to-TGN trafficking of CD8A fused furin and CI-M6PR. (a) Time course images showing the endocytic trafficking of CD8A-furin to Golgi. Mon2 or control siRNA treated HeLa cells were transfected to express CD8A-furin. Cells were surface labeled by CD8A antibody and chased for various lengths of time before immuno-labeling of giantin. In the panel “steady state”, total cellular CD8A-furin was labeled without pulse-chase. Bar, 10 μm. (b–c) Percentage of CD8A-furin and –CI-M6PR at Golgi in the time course of endocytic trafficking. n denotes the number of cells from 3 independent experiments. Error bar indicates standard error of mean. The p values (by t-test) of selected pair of data are indicated.

To explore the role of Mon2 in endocytic trafficking of furin and CI-M6PR, HeLa cells were first subjected to siRNA knockdown of Mon2 followed by transient expression of CD8A chimeras. Cells were subsequently incubated with an antibody against the extracellular domain of CD8A on ice to label the surface pool of CD8A chimera. After washing away the antibody, cells were chased at 37 °C for various lengths of time to allow endocytosis and post-endocytic trafficking. Under immunofluorescence, cells were imaged and the amount of chimera that reached the Golgi was quantified as the percentage of the giantin colocalized over the total CD8A chimera. A typical result of imaging and quantification for CD8A-furin is shown in Fig. 7a and b. At 0 min, CD8A-furin was located at the plasma membrane as fine puncta and co-localization with giantin was not observed (0 min). After 25 min of chase, CD8A-furin got internalized in both control and knockdown cells. In control cells, it became peripherally localized puncta, characteristic of endosomes. In contrast, in Mon2 knockdown cells, CD8A-furin had a perinuclear localization, which co-localized with giantin. Quantification indicated that the amount of CD8A transported to Golgi was significantly higher in knockdown than that of control cells (p = 0.005). The Golgi localized CD8A-furin remained much higher in Mon2 knockdown cells than control after 45 min of chase (p = 1 × 10^−7^). As the chase proceeded to 90 min, the amount of Golgi localized CD8A-furin in knockdown was less but still significantly more than the control (p = 0.05). CD8A-CI-M6PR showed a very similar trend (Fig. 7c and Sup. Fig. 5). Collectively, our data demonstrate that depletion of Mon2 accelerates the endocytic trafficking of furin and CI-M6PR from endosome to Golgi, therefore suggesting that Mon2 could normally suppress endosome-to-TGN trafficking or affect the localization and/or morphogenesis of endosome-TGN compartments.

At steady-state, we noted there was no difference in the amount of Golgi localized CD8A-CI-M6PR between control and knockdown cells (p = 0.59). However, in case of furin, significantly less TGN localized CD8A-furin was observed in knockdown cells at steady-state (p = 0.03). Since the TGN localized furin is determined by both inbound and outbound trafficking rate at Golgi, knockdown of Mon2 could promote trafficking at both directions but the outbound one could be boosted more than the endosome-to-TGN inbound trafficking.

## Discussion

Mon2/Ysl2 is conserved from yeast to human but its cellular function remains unclear. While most of our current knowledge of Mon2 is from yeast, its mammalian ortholog has yet to be characterized. In this study, we reported the first in depth characterization of mammalian Mon2. We first explored the sub-cellular localization of Mon2 in cultured mammalian cells. Consistent with the late Golgi localization of yeast Mon2p, in mammalian cells, endogenous or exogenously transfected Mon2 also localized to TGN. By serial truncations, we demonstrated that the Golgi localization of Mon2 requires both its N and C-termini. Our observation is different from the report in yeast, in which the N-terminal region of yeast Mon2p, including DCB and HUS domains, is necessary and sufficient for Golgi targeting^23^. The molecular mechanism behind the Golgi targeting of Mon2 is unknown. However, it is possible that such localization could be mediated by Arf1 signaling network since we observed that the Golgi association of Mon2 is very sensitive to BFA treatment similar to Arf1 effectors such as β-COP, AP1 and GGA2.

Arl1 is an important regulator for Golgi structure and function from yeast to mammal^9^,^11^. One of the key questions in this field is how Arl1 is regulated by its upstream factors, especially GEFs. While Syt1p could be a GEF for Arl1p in yeast^15^, the only known mammalian candidate for Arl1 GEF is Mon2. Three evidences supporting Mon2 as an Arl1 GEF were reported in yeast^21^. First, Mon2p is related to Arf GEFs in primary sequence and multiple alignment of Mon2p with Arf GEFs indicated a putative Sec7 domain; Second, Mon2p and Arl1p had genetic interaction; Third, the N-terminal fragment of Mon2p including the putative Sec7 region was able to pull down Arl1p in vitro. While recent studies confirmed that yeast Mon2p is homologous to GBF/BIG family Arf GEFs as they share domains such as DCB, HUS and HDS1-3, a significant Sec7 domain could not be identified in Mon2p with confidence using various bioinformatics tools^22^,^23^. Furthermore, deletion of yeast Mon2p didn't affect the Golgi localization of Arl1p, indicating that Mon2 is not required for Arl1 activation^11^,^22^. Finally, a recent finding suggested that Mon2 could be a negative regulator of Arl1p^27^. Therefore, Mon2 seems not to be a GEF for Arl1.

Here, we further addressed this issue in mammalian cells and our data suggested that mammalian Mon2 is neither necessary nor sufficient for the guanine nucleotide exchange of Arl1. First, we observed that Mon2 was much more sensitive toward BFA than Arl1 and its effectors. After 5 min of BFA treatment, Mon2 completely dissociated from Golgi apparatus. However, under the same condition, Golgin97 was still observed on Golgi, indicating the existence of Arl1-GTP on Golgi in the absence of Mon2. Second, we found that depletion of Mon2 didn't abolish or reduce the Golgi localization of Arl1 and its effectors. Third, the percentage of total active Arl1 was not affected by the depletion of Mon2. Together with previous studies from yeast^11^,^22^,^23^, our new data demonstrated that Mon2 is not essential for the guanine nucleotide exchange of Arl1. To test if Mon2 possesses GEF activity toward Arl1, we purified the N-terminal region of Mon2 including the putative Sec7 domain and subjected it to an in vitro guanine nucleotide exchange assay. While the Sec7 domain of BIG1 displayed a clear GEF activity for Arf1, the putative Sec7 domain of Mon2 did not exhibit significant exchange activity for Arl1 or Arf1. It is possible that the optimal GEF activity of Mon2 could require extra C-terminal regions and/or unknown factors that were not reconstituted in our in vitro assay. However, the simplest explanation of our data is that Mon2 is not a GEF for Arl1 or Arf1. Our data also suggested that GEFs for Arl1 could be different from those for Arfs as the Sec7 domain of BIG1 didn't have GEF activity toward Arl1 in vitro. The identity of the GEF for Arl1 in mammal remains open for further investigation.

Studies from yeast and worm all suggested that Mon2 could participate in the retrograde trafficking from endosome to Golgi^22^,^23^,^35^. Using CD8A fused furin and CI-M6PR reporters, we showed that mammalian Mon2 could also function in the same pathway. It is interesting to note that the depletion of Mon2 promoted the trafficking of furin and CI-M6PR to TGN, suggesting that Mon2 could normally slow down the retrograde trafficking pathway. The observation could not be easily explained by the known interaction partners of Mon2 – GGAs and Neo1p-Dop1p due to our limited knowledge of Mon2. Studies along this line could elucidate the cellular mechanism of Mon2. Since Mon2 and large Arf GEFs share many domains, knowledge of Mon2 should also facilitate our understanding of Arf GEFs.

## Methods

### Construction of DNA Plasmids

The C-terminus of human Mon2 including 3′-UTR from IMAGE clone 8860514 (Genbank Accession number: BC151241) was released by BglII/SacII digestion and ligated into pEGFP-C1 (Clontech) to obtain a Mon2 truncation clone 204–1718. The N-terminus of human Mon2 was PCR amplified using IMAGE clone 8860514 as template and the following primers: TAT AGA TCT ATG TCC GGC ACC AGC AGC and GCG AGA TCT TCT GTT ACT ATT TCC TTG. The resulting fragment was digested by BglII and ligated into the BglII digested Mon2 truncation clone 204–1718. The resulting clone with the correct orientation of the insert was the full length human Mon2 in pEGFP-C1. The double Myc-tagged full length Mon2 in pDMyc-neo vector^9^ was similarly cloned. Mon2 truncation clones 1–1035, 1–1200, 1–1333 and 1–1533 were cloned by conventional restriction digestions and PCRs.

To clone Sec7 domain of BIG1 in pGEB vector^6^, the Sec7 domain of human BIG1 was PCR amplified using HA-BIG1 (Martha Vaughan, National Institutes of Health) as template. To clone Δ14Arf1 in pET37b vector (Novogen), mouse Arf1 was PCR amplified using Arf1 in pGEB^6^ as template. To clone Δ14Arl1 in pET37b vector, rat Arl1 was PCR amplified using Arl1 in pGEB^6^ as template. Mon2 C-terminal fragment 1458–1711 was PCR amplified and inserted in pET37b vector for antigen production. To construct Mon2-N fragment for insect cell protein expression and guanine nucleotide exchange assay, an N-terminal fragment of Mon2 was PCR amplified by using Mon2 full length in pEGFP-C1 as template. The resulting PCR product was cloned into pFB-LIC-Bse vector using ligation independent cloning as previously described^39^.

To clone CD8A-furin chimera, the CD8A fragment was PCR amplified using a CD8A DNA plasmid as a template and the following oligonucleotides as primers: GTC TAG AAT TCA GCC ACC ATG GCC TTA CCA GTG ACC GCC TTG C (primer-CD8A-F) and CGA AAA CTA AAG CCA GAG CGC AGC TGG CAG TAA AGG GTG ATA ACC AGT G. The furin cytosolic domain fragment was PCR amplified by using IMAGE clone 6579931 (GenBank Acc No.: BU845991) as a template and the following oligonucleotides as primers: CAC TGG TTA TCA CCC TTT ACT GCC AGC TGC GCT CTG GCT TTA GTT TTC G and GAC CTG TCT AGA TCA GAG GGC GCT CTG GTC TTT GAT AAA GG (primer-furin-R). The two PCR fragments were mixed as template and subjected to PCR by primer-CD8A-F and primer-furin-R and the resulting product was digested by EcoRI/XbaI and ligated to pCI-neo vector (Promega) to yield CD8A-furin in pCI-neo. To clone CD8A-CI-M6PR chimera, the CD8A fragment was PCR amplified using the CD8A DNA plasmid as a template and the following oligonucleotides as primers: primer-CD8A-F and CAT TGT TTC CCT CCT CTT CTT CTT GCA GTA AAG GGT GAT AAC CAG TGA C. The cytosolic domain of CI-M6PR was PCR amplified by using IMAGE clone 6722707 (GenBank Accession No.: CA455182) as template and the following oligonculeotides as primers: GTC ACT GGT TAT CAC CCT TTA CTG CAA GAA GAA GAG GAG GGA AAC AAT G and GAC CTG TCT AGA TCA GAT GTG TAA GAG GTC CTC GTC (primer-CI-M6PR-R). The two fragments were mixed and subjected to PCR using the following primers: primer-CD8A-F and primer-CI-M6PR-R and the resulting product was digested by EcoRI/XbaI and ligated to pCI-neo vector (Promega) to yield CD8A-CI-M6PR in pCI-neo.

Except those indicated, PCR reactions were conducted by *Pfu* DNA polymerase and all clones were confirmed by sequencing. Golgin245 GRIP domain in pGEX-6P-1 was described previously^40^.

### Antibodies and chemicals

Mouse anti-GM130, Golgin245 and GGA2 monoclonal antibodies were from BD Transduction Laboratory (BD Bioscience). Rabbit anti-giantin and TGN46 polyclonal antibodies were from Abcam. Mouse anti-Golgin97 monoclonal antibody and Mant-GMPPNP were from (Molecular Probes). Mouse anti-GFP antibody was from Santa Cruz. OKT8 hybridoma culture medium was used as mouse anti-transferrin receptor antibody. Rabbit anti-Arl1 polyclonal antibody was described previously^9^. Brefeldin A (BFA) was from Epicenter.

### Cell culture, transfection and siRNA knockdown

HeLa and BSC1 cells were cultured in Dulbecco's Modified Eagle Medium supplemented with 10% fetal bovine serum. Transfection of plasmid DNA was conducted using Lipofectamine 2000 (Invitrogen) according to standard protocol. siRNA oligos were pre-designed from ThermoScientific. Two siRNA oligos targeting human Mon2 were: Y4 (GGCAGUGGGUCAACCUUUA) and Y5 (AAAUAUUGAUGUCGAGGUA). Control siRNA was GL2 (CGUACGCGGAAUACUUCGA). For 96-hour knockdown protocol, HeLa cells were transfected twice in 4 days using Lipofectamine 2000 (Invitrogen). The results shown were from Y5 siRNA though Y4 siRNA gave similar results.

### Mon2 antibodies

BL21 DE3 *E coli* bacteria were transformed by DNA plasmid Mon2 (1458–1711) in pET37b. The resulting bacteria were induced to express C-terminal His-tagged Mon2 (1458-1711) protein. The bacteria were pelleted and lysed by sonication in 8 M Urea phosphate buffered saline (PBS). After clearing the lysate by centrifugation, Ni-NTA agarose beads were incubated with the bacterial lysate for 2 hours at room temperature. The agarose beads were washed by 20 mM imidazole urea followed by elution in 200 mM imidazole urea. After dialysis in PBS, the precipitated fusion protein was used as antigen to inject New Zealand white rabbits. The resulting serum was collected and stored at −80 °C until use.

To purify polyclonal antibody against Mon2, nitrocellulose membrane was cut into pieces and subsequently incubated with the Mon2 (1458–1711)-His fusion protein in 8 M urea PBS to immobilize the antigen. After PBS washing, the membrane pieces were incubated with the serum at room temperature for 2 hours. The bound antibody was eluted by 50 mM glycine (pH2.5), neutralized, dialyzed, concentrated and quantified.

### Baculovirus and insect cell expression

Bacmid production, insect cell transfection and virus production were previously described^41^. The early passage of virus particles was amplified to passage 2 and used to infect 2 liters of log phase (3 × 10^6^ cells/ml) insect cells for the recombinant protein expression. The multiplicity of infection was kept between 2 to 3 and the culture was incubated at 140 rpm, 27°C for 56 hours.

### Active Arl1 pull-down assay

This was performed as described previously^30^.

### In vitro guanine nucleotide exchange assay

GST, GST-BIG1-Sec7, His-Δ14Arl1 and His-Δ14Arf1 proteins were purified from BL21 DE3 *E coli* bacteria transformed with DNA plasmid pGEB, BIG1-Sec7 in pGEB, Δ14Arl1 in pET37b and Δ14Arf1 in pET37b, respectively, as previously described^9^. GST and GST-BIG1-Sec7 were eluted from glutathione Sepharose beads (GE Health) by glutathione buffer (100 mM NaCl, 50 mM Tris pH 8.0, 10 mM reduced glutathione). His-Δ14Arl1 and His-Δ14Arf1 were eluted from Ni-NTA agarose beads (QIAGEN) by imidazole buffer (200 mM KCl, 20 mM HEPES, pH 7.3, 250 mM imidazole). Mon2-N was purified from insect sf9 cells essentially as previously described^39^. After Ni-NTA column and gel filtration, the fusion protein was subjected to Tobacco Etch Virus protease cleavage to remove the N-terminal His-tag followed by gel filtration. All fusion proteins were dialyzed against HK buffer (50 mM HEPES pH 7.5, 120 mM KCl). The exchange assay was conducted in a black 96-well plate (Greiner Bio-One). Each assay reaction had 150 μl HKM buffer (HK buffer supplemented with 1 mM MgCl_2_ and 1 mM DTT) containing 1 μM Mant-GMPPNP, 1 μM His-Δ14Arl1 or His-Δ14Arf1, and 1 μM EDTA or 0.7 μM testing protein (GST, GST-BIG1-Sec7 or Mon2-N). The fluorescence was monitored by Tecan Infinite M200 pro at 25 °C with the excitation at 360 nm and emission at 440 nm. The fluorescence data were collected every 30 or 50 seconds depending on the total number of samples per plate. The exchange kinetic traces with significant spikes were rejected. Single exponential curve fitting were analyzed in OriginPro 8.5 (Origin Lab).

### Immuofluorescence and microscopy

Immunofluorescence labeling was performed as described previously^6^. Cells were imaged by either an up-right laser scanning confocal microscope (Axioplan 2) equipped with LSM510 scanning head and 63× objective (Plan-apochromatic and N.A. 1.40) or an inverted wide-field microscope (Axiovert 200 M, Zeiss) with the following configurations–63× objective (Plan-apochromatic and N.A. 1.40), cooled charge coupled device camera (Coolsnap HQ, Photometrics) with a 0.65× adaptor lens and a Xenon lamp (X-cite, Lumen Dynamics).

### Morphological endocytic endosome-to-TGN transport assay

HeLa cells grown on glass coverslips were subjected to Mon2 or control siRNA knockdown for 96 hours. CD8A-furin or CD8A-CI-M6PR plasmid DNA was transfected one day before conducting the assay. At the end of the incubation, cells were first incubated with OKT8 hybridoma culture medium on ice. After washing away antibody by ice cold PBS, coverslips were incubated with tissue culture medium at 37°C in CO_2_ incubator for various lengths of time before fixation and immuno-labeling of giantin. The fluorescence images were acquired by a wide-field fluorescence microscope and analyzed in ImageJ (http://rsbweb.nih.gov/ij/). To quantify the amount of CD8A-furin or CI-M6PR in Golgi, a Golgi ROI (ROI_Golgi_) was generated by the segmentation of giantin signal. A ROI outlining the boundary of the cell (ROI_cell_) was manually drawn by tracing the cell contour aided by adjusting the brightness and contrast in CD8A channel. After background subtraction, the integrated intensities within ROI_Golgi_ and ROI_cell_ represented the relative amount of Golgi transported and internalized CD8A fusion proteins, respectively. The amount transported to Golgi was expressed as a percentage. T-test was performed in Microscoft Excel (two-tailed distribution and two-sample unequal variance).

## Author Contributions

L.L. designed the experiment scheme. D.M., B.K.B., Y.Z., L.C., L.L. performed experiments. T.C.C. prepared the purified Mon2-N fusion protein from inset cells. L.L., W.J.H. and D.M. analyzed data. L.L. and W.J.H. wrote the manuscript.

## Supplementary Material

Supplementary Information Mammalian Mon2/Ysl2 regulates endosome-to-Golgi trafficking but possesses no guanine nucleotide exchange activity toward Arl1 GTPase

## References

[b1] Gillingham A. K. & Munro S. The small G proteins of the Arf family and their regulators. Annu Rev Cell Dev Biol 23, 579–611 (2007). 17506703 10.1146/annurev.cellbio.23.090506.123209

[b2] D'Souza-Schorey C. & Chavrier P. ARF proteins: roles in membrane traffic and beyond. Nat Rev Mol Cell Biol 7, 347–358 (2006). 16633337 10.1038/nrm1910

[b3] Chardin P. *et al.* A human exchange factor for ARF contains Sec7- and pleckstrin-homology domains. Nature 384, 481–484 (1996). 8945478 10.1038/384481a0

[b4] Mouratou B. *et al.* The domain architecture of large guanine nucleotide exchange factors for the small GTP-binding protein Arf. BMC Genomics 6, 20 (2005). 15717927 10.1186/1471-2164-6-20 PMC553965

[b5] Kahn R. A. *et al.* Nomenclature for the human Arf family of GTP-binding proteins: ARF, ARL, and SAR proteins. J Cell Biol 172, 645–650 (2006). 16505163 10.1083/jcb.200512057 PMC2063696

[b6] Lu L., Horstmann H., Ng C. & Hong W. Regulation of Golgi structure and function by ARF-like protein 1 (Arl1). Journal of cell science 114, 4543–4555 (2001). 11792819 10.1242/jcs.114.24.4543

[b7] Lu L., Tai G. & Hong W. Autoantigen Golgin-97, an effector of Arl1 GTPase, participates in traffic from the endosome to the trans-golgi network. Mol Biol Cell 15, 4426–4443 (2004). 15269279 10.1091/mbc.E03-12-0872 PMC519138

[b8] Setty S. R., Shin M. E., Yoshino A., Marks M. S. & Burd C. G. Golgi recruitment of GRIP domain proteins by Arf-like GTPase 1 is regulated by Arf-like GTPase 3. Curr Biol 13, 401–404 (2003). 12620188 10.1016/s0960-9822(03)00089-7

[b9] Lu L. & Hong W. Interaction of Arl1-GTP with GRIP domains recruits autoantigens Golgin-97 and Golgin-245/p230 onto the Golgi. Molecular biology of the cell 14, 3767–3781 (2003). 12972563 10.1091/mbc.E03-01-0864 PMC196566

[b10] Liu Y. W., Huang C. F., Huang K. B. & Lee F. J. Role for Gcs1p in regulation of Arl1p at trans-Golgi compartments. Mol Biol Cell 16, 4024–4033 (2005). 15975906 10.1091/mbc.E05-01-0023 PMC1196316

[b11] Panic B., Whyte J. R. & Munro S. The ARF-like GTPases Arl1p and Arl3p act in a pathway that interacts with vesicle-tethering factors at the Golgi apparatus. Curr Biol 13, 405–410 (2003). 12620189 10.1016/s0960-9822(03)00091-5

[b12] Zahn C. *et al.* Knockout of Arfrp1 leads to disruption of ARF-like1 (ARL1) targeting to the trans-Golgi in mouse embryos and HeLa cells. Mol Membr Biol 23, 475–485 (2006). 17127620 10.1080/09687860600840100

[b13] Behnia R., Panic B., Whyte J. R. & Munro S. Targeting of the Arf-like GTPase Arl3p to the Golgi requires N-terminal acetylation and the membrane protein Sys1p. Nat Cell Biol 6, 405–413 (2004). 15077113 10.1038/ncb1120

[b14] Jones S. *et al.* Genetic interactions in yeast between Ypt GTPases and Arf guanine nucleotide exchangers. Genetics 152, 1543–1556 (1999). 10430582 10.1093/genetics/152.4.1543 PMC1460709

[b15] Chen K. Y. *et al.* Syt1p promotes activation of Arl1p at the late Golgi to recruit Imh1p. J Cell Sci 123, 3478–3489 (2010). 20841378 10.1242/jcs.074237

[b16] Wiederkehr A., Meier K. D. & Riezman H. Identification and characterization of Saccharomyces cerevisiae mutants defective in fluid-phase endocytosis. Yeast 18, 759–773 (2001). 11378903 10.1002/yea.726

[b17] Muren E., Oyen M., Barmark G. & Ronne H. Identification of yeast deletion strains that are hypersensitive to brefeldin A or monensin, two drugs that affect intracellular transport. Yeast 18, 163–172 (2001). 11169758 10.1002/1097-0061(20010130)18:2<163::AID-YEA659>3.0.CO;2-#

[b18] Avaro S., Belgareh-Touze N., Sibella-Arguelles C., Volland C. & Haguenauer-Tsapis R. Mutants defective in secretory/vacuolar pathways in the EUROFAN collection of yeast disruptants. Yeast 19, 351–371 (2002). 11870858 10.1002/yea.838

[b19] Bonangelino C. J., Chavez E. M. & Bonifacino J. S. Genomic screen for vacuolar protein sorting genes in Saccharomyces cerevisiae. Mol Biol Cell 13, 2486–2501 (2002). 12134085 10.1091/mbc.02-01-0005 PMC117329

[b20] Singer-Kruger B. & Ferro-Novick S. Use of a synthetic lethal screen to identify yeast mutants impaired in endocytosis, vacuolar protein sorting and the organization of the cytoskeleton. Eur J Cell Biol 74, 365–375 (1997). 9438133

[b21] Jochum A., Jackson D., Schwarz H., Pipkorn R. & Singer-Kruger B. Yeast Ysl2p, homologous to Sec7 domain guanine nucleotide exchange factors, functions in endocytosis and maintenance of vacuole integrity and interacts with the Arf-Like small GTPase Arl1p. Mol Cell Biol 22, 4914–4928 (2002). 12052896 10.1128/MCB.22.13.4914-4928.2002 PMC133889

[b22] Gillingham A. K., Whyte J. R., Panic B. & Munro S. Mon2, a relative of large Arf exchange factors, recruits Dop1 to the Golgi apparatus. J Biol Chem 281, 2273–2280 (2006). 16301316 10.1074/jbc.M510176200

[b23] Efe J. A. *et al.* Yeast Mon2p is a highly conserved protein that functions in the cytoplasm-to-vacuole transport pathway and is required for Golgi homeostasis. J Cell Sci 118, 4751–4764 (2005). 16219684 10.1242/jcs.02599

[b24] Barbosa S., Pratte D., Schwarz H., Pipkorn R. & Singer-Kruger B. Oligomeric Dop1p is part of the endosomal Neo1p-Ysl2p-Arl1p membrane remodeling complex. Traffic 11, 1092–1106 (2010). 20477991 10.1111/j.1600-0854.2010.01079.x

[b25] Wicky S., Schwarz H. & Singer-Kruger B. Molecular interactions of yeast Neo1p, an essential member of the Drs2 family of aminophospholipid translocases, and its role in membrane trafficking within the endomembrane system. Mol Cell Biol 24, 7402–7418 (2004). 15314152 10.1128/MCB.24.17.7402-7418.2004 PMC507011

[b26] Singer-Kruger B. *et al.* Yeast and human Ysl2p/hMon2 interact with Gga adaptors and mediate their subcellular distribution. EMBO J 27, 1423–1435 (2008). 18418388 10.1038/emboj.2008.75 PMC2396392

[b27] Manlandro C. M. *et al.* Mon2 is a negative regulator of the monomeric G protein, Arl1. FEMS Yeast Res 12, 637–650 (2012). 22594927 10.1111/j.1567-1364.2012.00814.x

[b28] Lippincott-Schwartz J., Roberts T. H. & Hirschberg K. Secretory protein trafficking and organelle dynamics in living cells. Annu Rev Cell Dev Biol 16, 557–589 (2000). 11031247 10.1146/annurev.cellbio.16.1.557 PMC4781643

[b29] Boman A. L. GGA proteins: new players in the sorting game. J Cell Sci 114, 3413–3418 (2001). 11682601 10.1242/jcs.114.19.3413

[b30] Lu L., Tai G. & Hong W. Interaction of Arl1 GTPase with the GRIP domain of Golgin-245 as assessed by GST (glutathione-S-transferase) pull-down experiments. Methods Enzymol 404, 432–441 (2005). 16413289 10.1016/S0076-6879(05)04038-3

[b31] Morinaga N., Adamik R., Moss J. & Vaughan M. Brefeldin A inhibited activity of the sec7 domain of p200, a mammalian guanine nucleotide-exchange protein for ADP-ribosylation factors. J Biol Chem 274, 17417–17423 (1999). 10364170 10.1074/jbc.274.25.17417

[b32] Kahn R. A. *et al.* The amino terminus of ADP-ribosylation factor (ARF) is a critical determinant of ARF activities and is a potent and specific inhibitor of protein transport. J Biol Chem 267, 13039–13046 (1992). 1618801

[b33] Antonny B., Beraud-Dufour S., Chardin P. & Chabre M. N-terminal hydrophobic residues of the G-protein ADP-ribosylation factor-1 insert into membrane phospholipids upon GDP to GTP exchange. Biochemistry 36, 4675–4684 (1997). 9109679 10.1021/bi962252b

[b34] Richardson B. C., McDonold C. M. & Fromme J. C. The Sec7 Arf-GEF is recruited to the trans-Golgi network by positive feedback. Dev Cell 22, 799–810 (2012). 22516198 10.1016/j.devcel.2012.02.006 PMC3331996

[b35] Kanamori T. *et al.* Beta-catenin asymmetry is regulated by PLA1 and retrograde traffic in C. elegans stem cell divisions. EMBO J 27, 1647–1657 (2008). 18497747 10.1038/emboj.2008.102 PMC2396877

[b36] Seaman M. N. Cargo-selective endosomal sorting for retrieval to the Golgi requires retromer. J Cell Biol 165, 111–122 (2004). 15078902 10.1083/jcb.200312034 PMC2172078

[b37] Lin S. X., Mallet W. G., Huang A. Y. & Maxfield F. R. Endocytosed cation-independent mannose 6-phosphate receptor traffics via the endocytic recycling compartment en route to the trans-Golgi network and a subpopulation of late endosomes. Mol Biol Cell 15, 721–733 (2004). 14595110 10.1091/mbc.E03-07-0497 PMC329388

[b38] Mallet W. G. & Maxfield F. R. Chimeric forms of furin and TGN38 are transported with the plasma membrane in the trans-Golgi network via distinct endosomal pathways. J Cell Biol 146, 345–359 (1999). 10465644 10.1083/jcb.146.2.345 PMC2156176

[b39] Graslund S. *et al.* Protein production and purification. Nat Methods 5, 135–146 (2008). 18235434 10.1038/nmeth.f.202 PMC3178102

[b40] Wu M., Lu L., Hong W. & Song H. Structural basis for recruitment of GRIP domain golgin-245 by small GTPase Arl1. Nat Struct Mol Biol 11, 86–94 (2004). 14718928 10.1038/nsmb714

[b41] Shrestha B., Smee C. & Gileadi O. Baculovirus expression vector system: an emerging host for high-throughput eukaryotic protein expression. Methods Mol Biol 439, 269–289 (2008). 18370110 10.1007/978-1-59745-188-8_19

