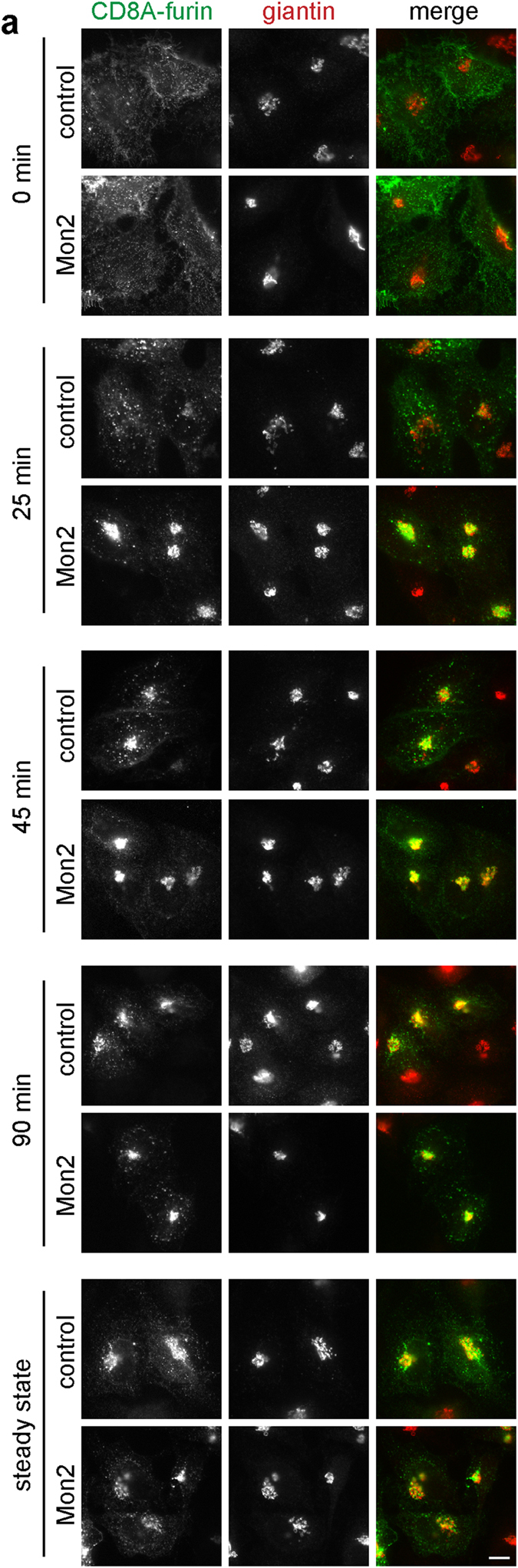# Corrigendum: Mammalian Mon2/Ysl2 regulates endosome-to-Golgi trafficking but possesses no guanine nucleotide exchange activity toward Arl1 GTPase

**DOI:** 10.1038/srep33619

**Published:** 2016-09-22

**Authors:** Divyanshu Mahajan, Boon Kim Boh, Yan Zhou, Li Chen, Tobias Carl Cornvik, Wanjin Hong, Lei Lu

Scientific Reports 3: Article number: 0336210.1038/srep03362; published online: 11 28 2013; updated: 09 22 2016

This Article contains an error in Figure 7a. The image depicting CD8A-furin ‘90 min, Mon2’ is a duplicate of the image depicting CD8A-furin ‘steady state, Mon2’. The Figure legend is correct. The correct Figure 7a appears below as Figure 1.

## Figures and Tables

**Figure 1:**