# Supplementary material for: Mammalian Mon2/Ysl2 regulates endosome-to-Golgi trafficking but possesses no guanine nucleotide exchange activity toward Arl1 GTPase
Source: Sci Rep. 2013 Nov 28;3:3362. doi: 10.1038/srep03362 (PMC3842536; doi:10.1038/srep03362)
Supplement: Supplementary Information — Mammalian Mon2/Ysl2 regulates endosome-to-Golgi trafficking but possesses no guanine nucleotide exchange activity toward Arl1 GTPase [file srep03362-s1.pdf]

Mammalian Mon2/Ysl2 regulates endosome-to-Golgi trafficking but possesses no guanine nucleotide exchange activity toward Arl1 GTPase

Divyanshu Mahajan[1], Boon Kim Boh[1], Yan Zhou[1], Li Chen[1], Tobias Carl Cornvik[1], Wanjin Hong[2] and Lei Lu[1,*]

[1]School of Biological Sciences, Nanyang Technological University, 60 Nanyang Drive, Singapore 637551

[2]Institute of Molecular and Cell Biology, 61 Biopolis Drive, Proteos, Singapore 138673

*Corresponding author

Corresponding author

Lei Lu

SBS-03n-18

School of Biological Sciences

Nanyang Technological University

60 Nanyang Drive

Singapore 637551

Email: lulei@ntu.edu.sg

Tel: (65) 6592-2591

Fax: (65) 6791-3856

Supplementary information

Supplementary Figure 1 Characterization of Mon2 polyclonal antibody. (a) Mon2 polyclonal antibody is able to detect both endogenous and transfected Mon2. Cytosol was extracted from plain or 293 cells transiently expressing either Myc-Mon2 or GFP-Mon2 using a buffer containing 40 mM HEPES pH 7.3, 100 mM NaCl, 0.1% Triton X-100. The resulting extracts were resolved in gel and blotted by Mon2 polyclonal antibody. The image was cropped to highlight the region of interest and the original gel blot is presented in Sup. Fig. 6c. (b) Mon2 polyclonal antibody is able to immunoprecipitate endogenous Mon2. Cytosol was extracted as above and incubated with Mon2 polyclonal antibody for 4 hours at 4 °C. The antibody was captured by Protein A Sepharose beads (GE Health) for 2 hours at 4 °C. After washing, the bead bound protein was eluted by SDS-sample buffer and detected in Western blot by Mon2 polyclonal antibody. The image was cropped to highlight the region of interest and the original gel blot is presented in Sup. Fig. 6d. (c) Mon2 antigen specifically blocks the Golgi staining of Mon2 antibody. Mon2 polyclonal antibody was incubated with its antigen, which was immobilized on nitrocellulose membrane pieces. The resulting supernatant was used to stain HeLa cells together with anti-GM130 antibody as in Fig. 1a. (d) The perinuclear Golgi staining by Mon2 antibody was specifically abolished after Mon2 knockdown. Control or Mon2 siRNA incubated HeLa cells were stained by Mon2 polyclonal antibody. (e) Myc-Mon2 localized to Golgi in NRL cells. NRL cells were transiently transfected by Myc-Mon2 and co-stained by anti-Myc (9E10) and anti-Arl1 antibody. Bars, 10 µm.

Supplementary Figure 2 Both Mon2 and GGA2 dissociate from Golgi in less than 5 min of BFA treatment. HeLa cells were treated with 10 µg/ml BFA for 0 min (upper panel), 5 min (middle panel) and 10 min (lower panel). Cells were labeled by antibodies against Mon2 and GGA2. Bar, 10 µm.

Supplementary Figure 3 Localization of wild type and truncated EGFP-Mon2. BSC1 cells transfected with EGFP-Mon2 wild type (upper panel), truncation clones 1-1035 (middle panel) and 1-1200 (lower panel) were labeled by anti-GM130 antibody. Bar, 10 μm.

Supplementary Figure 4 In vitro guanine nucleotide exchange assay. (a) Coomassie stained SDS-PAGE showing purified GST, GST-BIG1-Sec7, His-Δ14Arl1 and His-Δ14Arf1. (b) Typical exchange kinetic traces during in vitro guanine nucleotide exchange of Arf. Plots are similarly labeled as in Fig. 6b-f.

Supplementary Figure 5 Depletion of Mon2 accelerates the endosome-to-TGN trafficking of CD8A fused CI-M6PR. Images are similarly organized and labeled as in Fig. 7a.

Supplementary Figure 6 Original gel blots before cropping. Western blot images were acquired by a cooled CCD camera. The gel blots were trimmed according to molecular weights to reduce the antibody blotting volumes or to probe multiple proteins. The regions of interest are highlighted in boxes. (a) The original gel blots for Fig. 4a. The upper and lower parts of the gel blot were probed by anti-Mon2 and anti-tubulin antibody, respectively. (b) The original gel blot for Fig. 5a. (c-d) The original gel blots for Sup. Fig. 1a and b.

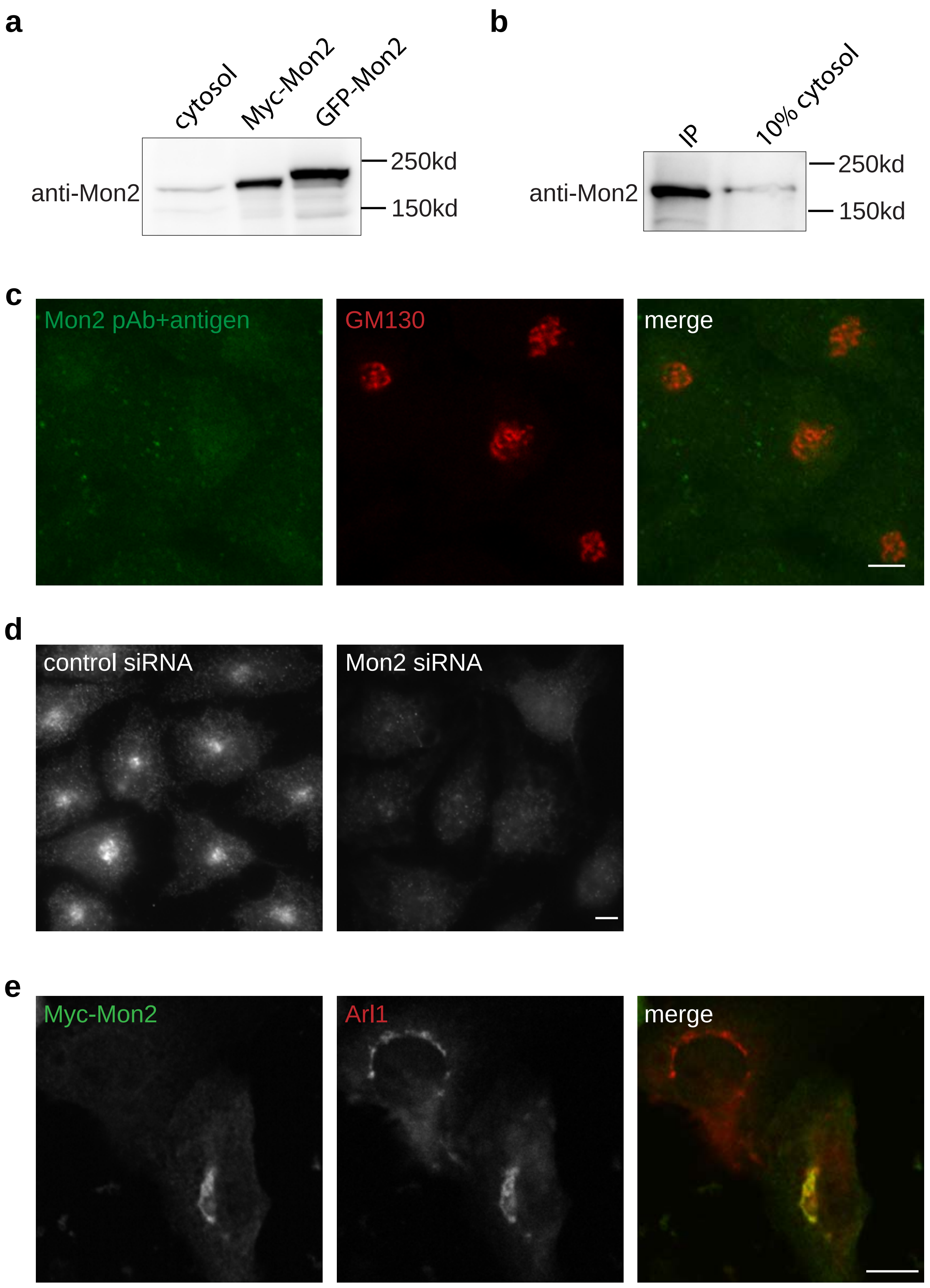

Supplementary Fig. 1

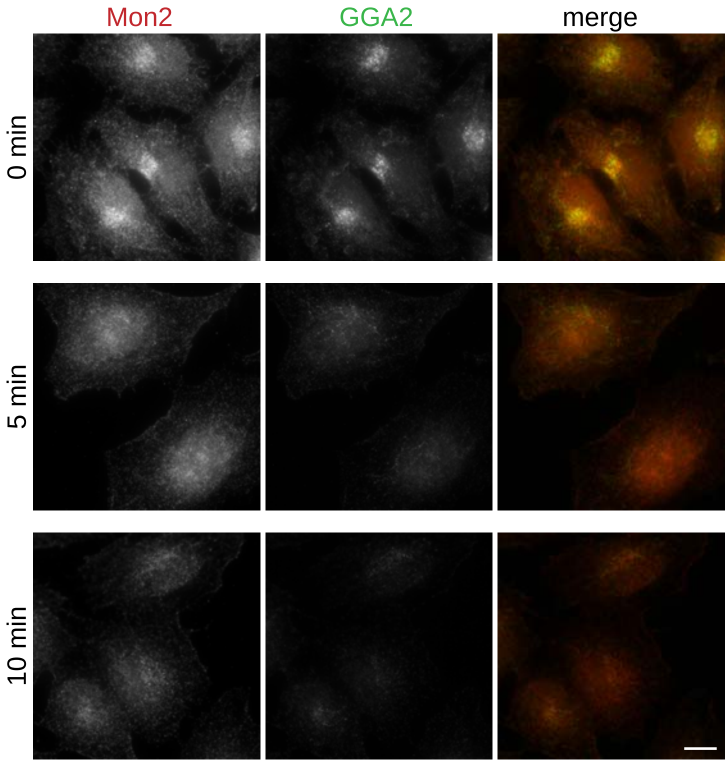

Supplementary Fig. 2

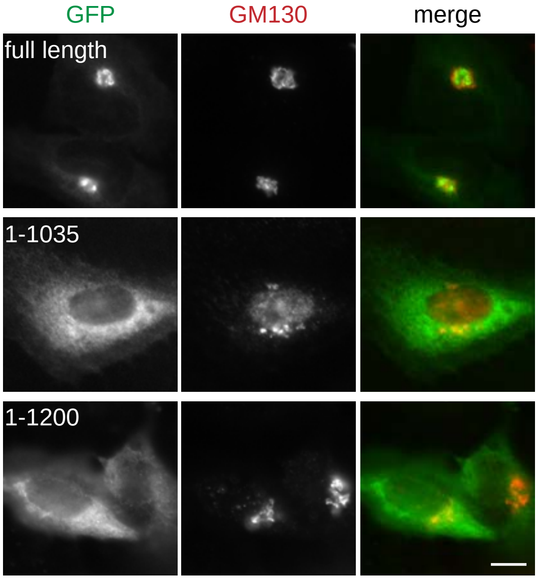

Supplementary Fig. 3

a

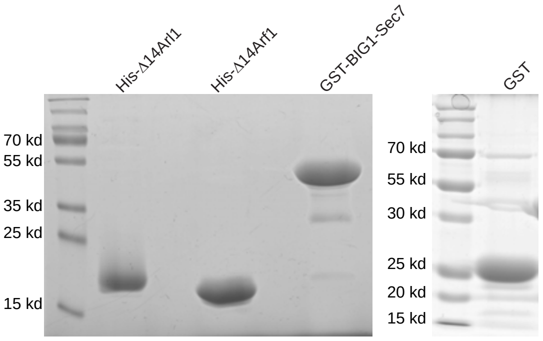

b

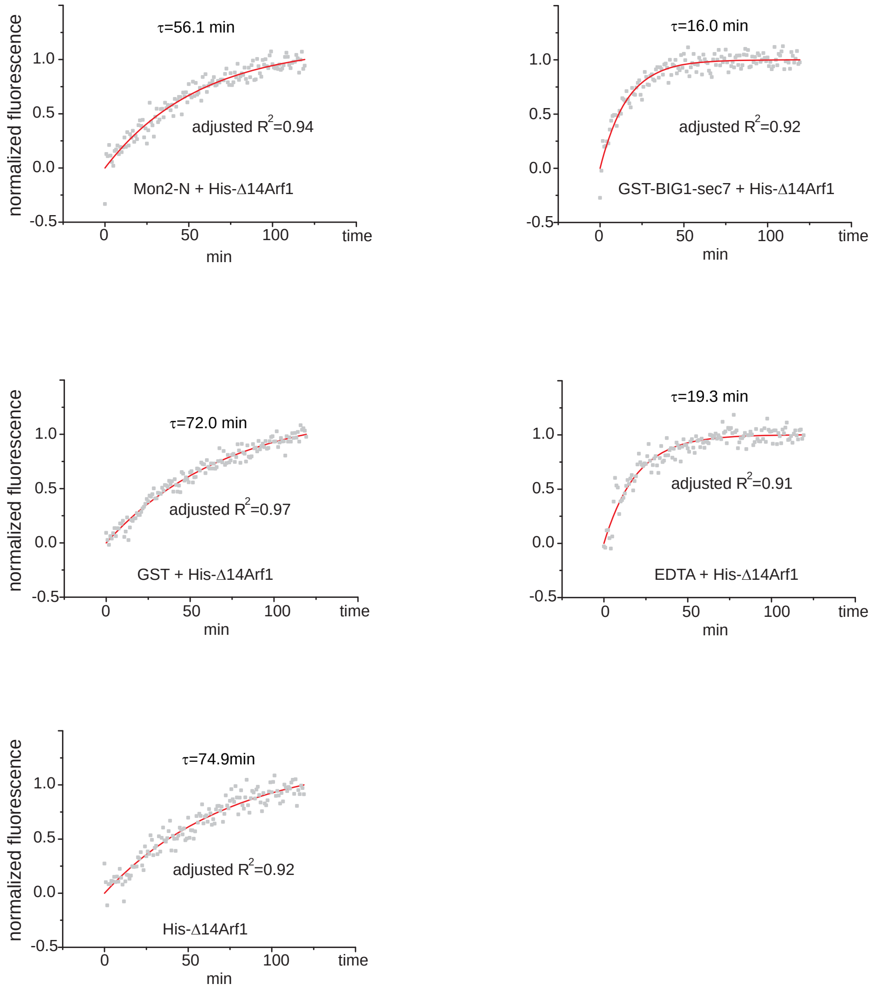

Supplementary Fig. 4

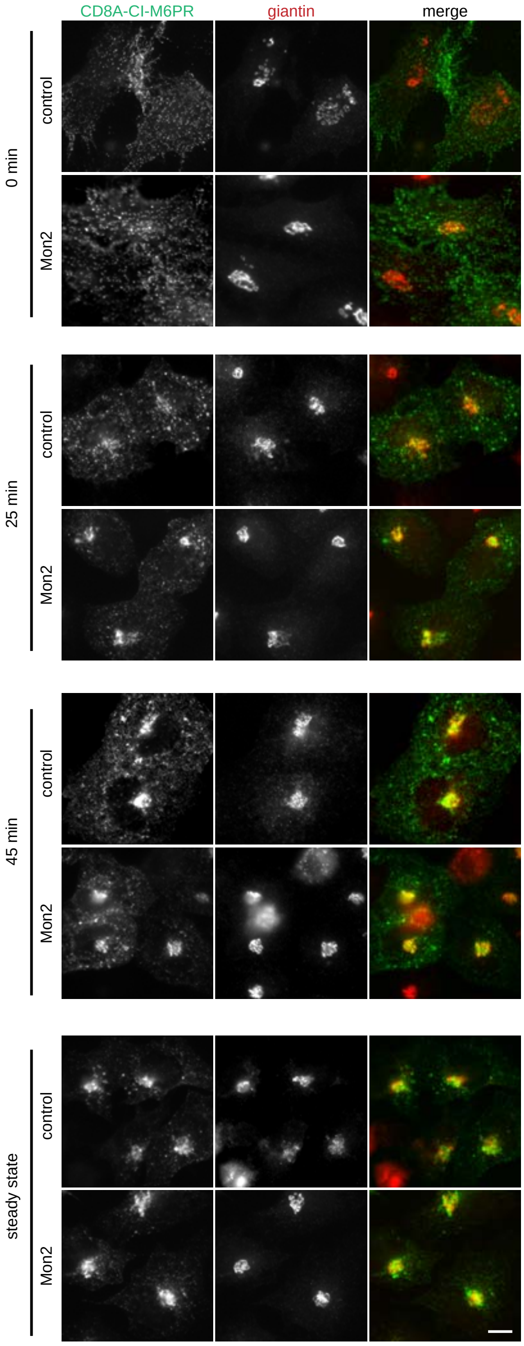

Supplementary Fig. 5

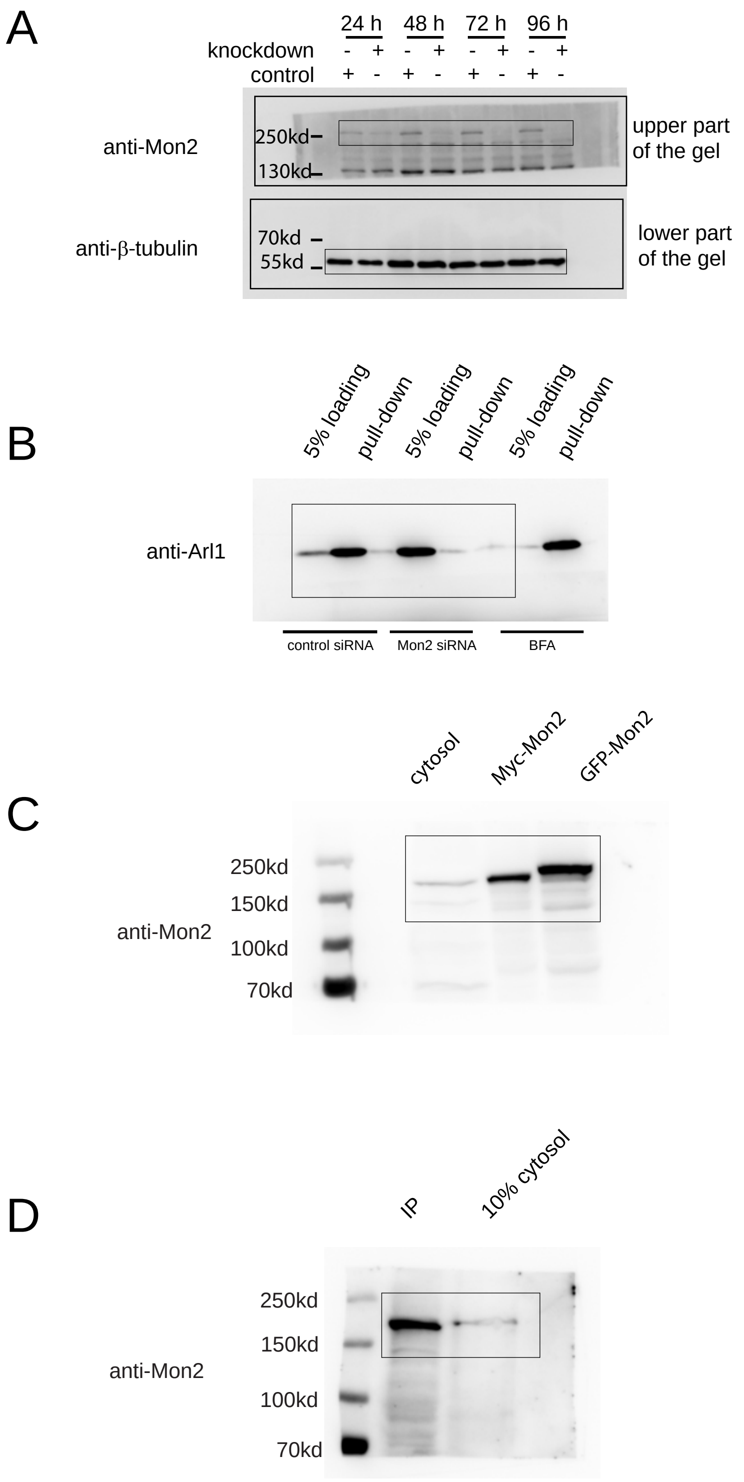

Supplementary Fig. 6
